# Underlying conditions contributing to breathlessness in the population

**DOI:** 10.1097/SPC.0000000000000568

**Published:** 2021-10-04

**Authors:** Jacob Sandberg, Max Olsson, Magnus Ekström

**Affiliations:** Department of Clinical Sciences, Division of Respiratory Medicine & Allergology, Lund University, Lund, Sweden

**Keywords:** breathlessness, causes, general population, underlying conditions

## Abstract

**Purpose of review:**

Assessment of underlying conditions that contribute to breathlessness is fundamental for symptom management. This review aims to summarize the knowledge from the past two years on the most common underlying conditions among individuals with breathlessness in the general population and to identify research gaps.

**Recent findings:**

Nine studies from the last two years were included in the review; two studies systematically assessed underlying conditions among breathless individuals in the general population. The modified Medical Research Council (mMRC) scale was used in eight of nine studies. Respiratory diseases were the main underlying condition (40–57%), of which asthma was the most common (approx. 25%), and chronic obstructive pulmonary disease was particularly strongly associated with breathlessness. Other conditions prevalent among breathless individuals included heart diseases, anxiety, depression, and obesity, and several conditions often co-existed.

**Summary:**

Breathlessness in the general population is common and associated with several underlying conditions. Respiratory disease is the most commonly reported underlying condition. Refined methods such as machine learning could be useful to study the complex interplay between multiple underlying causes of breathlessness and impact on outcomes such as quality of life and survival.

## INTRODUCTION

Breathlessness is a common symptom in the general population. The prevalence is increasing with age and is reported to be 10–25% among middle-aged and older individuals [1–3]. It is associated with many of the most common diseases, such as chronic obstructive pulmonary disease (COPD) and heart failure [4]. Individuals with breathlessness have increased morbidity and mortality, lower quality of life and increased healthcare utilization [4–6]. The severity of breathlessness often increases in advanced stages of diseases and at the end of life [7].

The pathophysiology of breathlessness involves several mechanisms. For example, an increased work of breathing through stimulation of receptors in the airways, lung parenchyma and/or the chest wall or by stimulation of central and peripheral chemoreceptors. This leads to increased muscle work that is further influenced by the current affective and mental state [8,9].

In heart failure, breathlessness is caused by increased pressure in the capillary bed and pulmonary congestion due to the reduced heart function [10]. For obesity it has been suggested that breathlessness develops from the increased workload and the resulting respiratory demand [11,12]. Anxiety and/or depression is more common among individuals with breathlessness and is thought to also cause and worsen breathlessness through reduced gating of respiratory sensory information in the brain and heightened breathing awareness [13].

Identification of underlying conditions is a fundamental part of clinical evaluation and management of breathlessness. The information is needed to guide optimal management [14].

The most common reported underlying condition among breathless individuals has been respiratory disease, heart disease and obesity [1,3,15]. Increased comorbidity is associated with increased breathlessness [1] and COPD is known to often co-exist with heart disease as both conditions share risk factors [16].

Knowledge is limited on which conditions contribute to breathlessness, as well as the level of overlap between conditions in the general population. Many studies have focused on one disease or population only. The challenges when studying and evaluating breathlessness includes the presence of complex associations between multiple factors that contribute to the sensation, including heart and lung diseases, different mental states, hereditary factors, and social and environmental factors [17]. Many of these factors often coexist [18], and methods capable of analysing complex interplays between concurrent factors are needed to gain a fuller understanding of breathlessness.

Machine learning has emerged as a modern alternative to regular statistical methods for analysing datasets with large number of variables. Machine learning consists of a group of algorithms that learns to make predictions from data [19], for example, to classify an outcome of a health conditions using a large number of clinical variables. Machine learning also offers great possibilities to rank and compare the importance of different factors for an outcome [20] such as breathlessness. This variable importance can be used to identify complex, nonlinear associations to the outcome that may not have been identified with regular regression methods.

The aim of this study was to review the recent knowledge on the epidemiology of underlying conditions among breathless individuals in the general population. The focus was on papers published in the last two years and an additional aim was to identify knowledge gaps for future studies. 

**Box 1:**
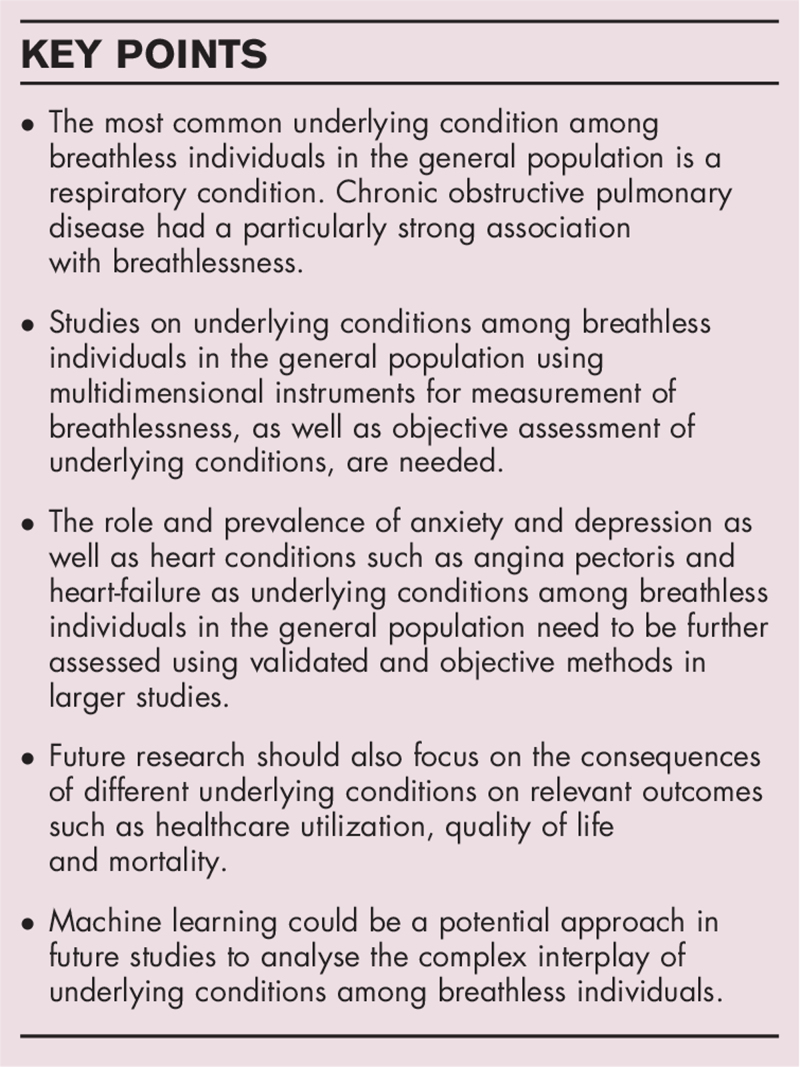
no caption available

## SEARCH STRATEGY

**FIGURE 1:**
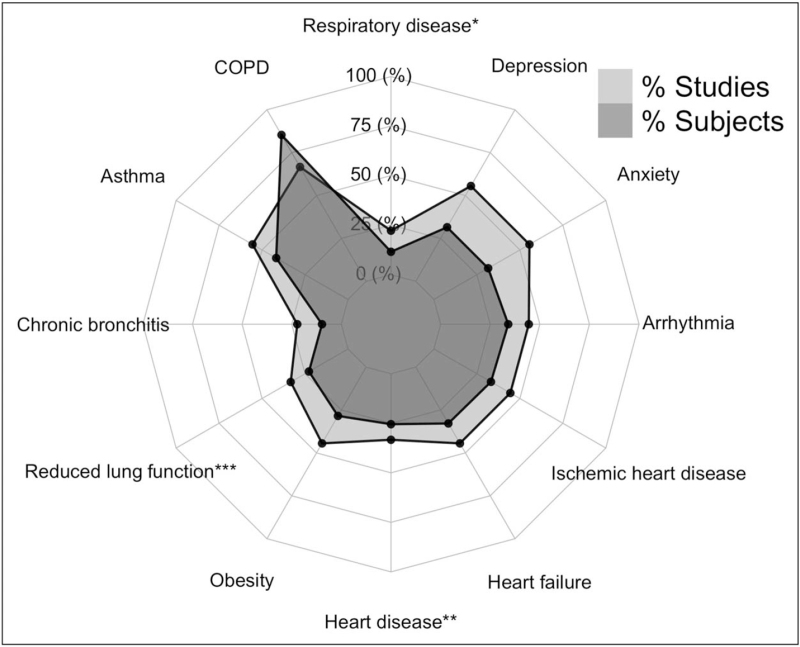
Percentage of studies (*n* = 9) and included subjects (*n* = 96 614) in analyses of association between underlying conditions and breathlessness. Further from the centre represents a higher percentage. The plot presents a percentage of studies and subjects that evaluate the association between the conditions and breathlessness, which does not necessarily mean a significant association. ^∗^Studies with aggregated data on respiratory diseases. ^∗∗^Studies with aggregated data on heart diseases. ^∗∗∗^Studies with data on reduced lung function from spirometry examinations.

We searched Medline from June 04, 2019 up to June 04, 2021 using the search terms (‘breathlessness OR dyspnea OR dyspnea OR shortness of breath OR breathing difficulty’ AND ‘general population’). Searches were restricted to adults and to articles in English. Reference lists of identified articles and personal libraries were searched, including for articles in press. Inclusion criteria were that the article provided sufficient quantitative data on breathless individuals and underlying conditions with relevance for the middle-aged and elderly general population. The initial search yielded 525 reports which were screened manually based on title and abstract (by JS and MO). Conflicting judgements were resolved through consensus discussion with the third author (ME). A total of 45 articles were selected for full text review. Of these, 36 were excluded: 16 due to not having data on contributing conditions to breathlessness, 13 for having the wrong patient population (being disease specific) and seven due to focusing on specific study populations (specific disease or patient group). A final nine studies that fulfilled the inclusion criteria were included in the review (Fig. 1).

## RECENT FINDINGS

### Overview of identified studies

Of the included nine studies, eight were cross-sectional [21,22^▪^,23^▪^,^24^,^25^,^26^–^28^^▪▪^] and one was a longitudinal cohort study [29]. Breathlessness was measured using the modified Medical Research Council (mMRC) in eight of the nine studies [22^▪^,23^▪^,^24^,^25^,26^▪^–28^▪▪^,^29^]. The remaining study [21] used a yes/no question on having breathlessness ‘when hurrying or climbing the stairway’. Breathlessness was defined as mMRC ≥1 in four studies [22^▪^,^24^,27^▪^,^28^^▪▪^] and as mMRC ≥2 [23^▪^,^25^,26^▪^,^29^] in the others. For the purpose of this review, presence of breathlessness was defined as mMRC ≥1 unless otherwise specified. Underlying conditions were self-reported by the participants in all studies, but some studies obtained additional data from spirometry [24,25,27^▪^,^28^^▪▪^,^29^], echocardiography (ECG) [27^▪^] exercise testing [27^▪^] and weight and height measurements [27^▪^,^28^^▪▪^]. The largest study included 43 271 individuals [24], the second largest 13 437 [28^▪▪^], and the other seven studies had population samples of fewer than 10 000 individuals [21,25,26^▪^,^29^]. The smallest study had data on 1097 individuals [27^▪^]. Slightly different age groups were studied, 50–64 years in three of the studies [27^▪^,^28^^▪▪^,^29^], older than 40 years in one study [25] and all adult age groups (>18 years) were included in five studies [21,22^▪^,23^▪^,^24^,26^▪^].

### Contributing causes of breathlessness in the general population

Two studies reported systematic evaluations of underlying conditions among breathlessness individuals as a primary aim [26^▪^,27^▪^]. The most common reported underlying condition was respiratory disease [26^▪^,27^▪^], but heart disease [25,26^▪^–28^▪▪^], anxiety [23^▪^,26^▪^,27^▪^], depression [23^▪^,26^▪^,27^▪^], and obesity [26^▪^–28^▪▪^] were also common among individuals with breathlessness. The most common conditions contributing to breathlessness are reviewed in more detail below.

### Respiratory diseases

A study by Sandberg *et al.* (*n* = 1097) [27^▪^] found that 57% of individuals reporting breathlessness had one or more respiratory disease, defined as any of asthma, COPD, chronic bronchitis, other respiratory disease, or a spirometry pattern of either chronic airflow limitation or restriction. Another population study by Poulos *et al.* (*n* = 10 072) [26^▪^] found that 40% of breathless individuals reported receiving a respiratory diagnosis from a doctor and having that condition at present including any of COPD, asthma, chronic bronchitis, emphysema, bronchiectasis, pneumonia, tuberculosis, silicosis, pulmonary fibrosis, asbestosis, pneumothorax, or lung cancer. Asthma was the most common respiratory condition (25% and 24%) in both studies and COPD was reported in 22% and 11.2% of people with breathlessness, respectively [26^▪^,27^▪^].

The association with having breathlessness was consistently stronger for COPD than for asthma [24,26^▪^,27^▪^,^29^]; the odds ratio (OR) for having breathlessness was 7.4 (95% confidence interval [CI]: 3.0–18.5) for COPD and OR 3.0 (95% CI: 1.7–5.2) for asthma [27^▪^]. The association with breathlessness measured as risk ratio (RR) was also found to be stronger for COPD (RR 4.2; 95% CI: 3.7–4.8) than for asthma (RR 2.6; 95% CI: 2.3–3.0) [26^▪^].

Chronic bronchitis was shown to be present in 20% of individuals reporting breathlessness and to be strongly associated with breathlessness, OR 3.6 (95% CI: 2.0–6.6) [27^▪^] and OR 2.7 (95% CI: 1.4–5.5) [21].

Lung function in relation to breathlessness in the general population was only reported in a few studies. One study reported reduced lung function on spirometry in 35% of individuals with breathlessness compared to in 13% in those without breathlessness [29]. Chronic airflow limitation (defined as post bronchodilator forced expiratory volume in 1 s [FEV_1_]/forced vital capacity [FVC]) below the fifth percentile) was present in 23% of those with breathlessness [27^▪^]. Restriction on static spirometry (defined as a total lung capacity below the fifth percentile) was present among 9% of breathless individuals, but the number of cases were few (*n* = 9) and the study was unable to detect any statistically significant difference from the group without breathlessness [27^▪^].

### Heart disease

The prevalence of heart disease among breathless individuals in the general population was 35%, 23% and 12% in the studies reporting percentages [26^▪^–28^▪▪^]. The definition of heart disease comprised a history of heart failure, arrhythmia, or heart attack. Two of the studies included the presence of angina pectoris in the definition [26^▪^,27^▪^], possibly explaining some of the between study difference in rates.

Heart failure was present in 4.9% [26^▪^], 3.8% [27^▪^] and 3.7% [28^▪▪^] of individuals with breathlessness. Associations between heart failure and breathlessness varied substantially, with adjusted ORs of 6.6 (95% CI: 1.3–33.3) for mMRC ≥1 [27^▪^], 21.6 (95% CI: 11.2–41.7) for mMRC = 2 [25], 15.0 (95% CI: 6.2–36.7) for mMRC ≥2 [25] and finally a RR of 3.4 (95% CI: 2.7–3.2) for mMRC ≥ 2 [26^▪^]. In two of these studies the number of people with heart failure was small resulting in high uncertainty of the results as demonstrated by large 95% confidence intervals [25,27^▪^].

Previous heart attack or coronary heart disease, including signs of angina pectoris, were reported among 15.5% of breathless individuals [26^▪^] and associated with breathlessness (RR 2.7; 95% CI: 2.3–3.1). Coronary heart disease without adding angina pectoris was present in 4% [27^▪^] and 6% [28^▪▪^] of cases. Angina pectoris only was present in 30% of the individuals with breathlessness and associated with breathlessness (adjusted OR 9.3; 95% CI: 5.1–17.2) [27^▪^].

Arrythmias, assessed through self-report, were present among 11% (*n* = 110) of breathless individuals and was associated with having breathlessness in the population (RR 2.3; 95% CI: 2.0–2.8) [26^▪^]. Atrial fibrillation based on ECG findings only was present among 2% (*n* = 2) of breathless individuals and not statistically significant from the group without breathlessness (*P* = 0.19) [27^▪^].

### Anxiety and/or depression

Anxiety and/or depression were evaluated using different methods in different studies. One study used the Patient Health Questionnaire-4 [23^▪^], two studies used the short form Composite International diagnostic Interview for the diagnostic and Statistical Manual of Mental disorders [27^▪^,^28^^▪▪^] and the fourth study used self-report (yes/no) of anxiety or depression [26^▪^].

Anxiety and depression were common among individuals with breathlessness with reported prevalence at 52% [27^▪^], 48% [26^▪^] and 26% [23^▪^] from different studies. Increasing anxiety was associated with breathlessness [22^▪^]; OR 1.8 (95% CI: 1.0–3–0) for mMRC = 1, OR 4.9 (95% CI: 2.2–10.9) for mMRC = 2 and OR 7.2 (95% CI: 3.8–13.7) for mMRC 3–4.

In one study, 8% had anxiety only and 10% had depression only [23^▪^] whereas two other studies (with different populations but from the same setting and using the same method for categorization) showed that approximately 36% had anxiety and 31–33% had depression but the level of overlap was unclear [27^▪^,^28^^▪▪^]. Depression may have a stronger association (OR 3.3; 95% CI: 2.0–5.4) with breathlessness than anxiety (OR 2.2; 95% CI: 1.4–3.5) but the difference was not statistically significant [27^▪^].

### Obesity

Three studies during the review period assessed the relationship between obesity and breathlessness in the general population [26^▪^–28^▪▪^]. All three studies defined obesity as having a body mass index (BMI >30 kg/m^2^. One study also included data on being overweight defined as a BMI between 25 and 30 [28^▪▪^].

Obesity was present among approximately 43% of the breathless individuals in two of the studies (defined as mMRC ≥ 1 in one study) (*n* = 1097) [27^▪^] and mMRC ≥2 in the other (*n* = 10 037) [26^▪^]) and among 42% in the third (*n* = 13 437) [28^▪▪^]. Thirty-five percent of breathless individuals were overweight [28^▪▪^].

Associations between obesity and breathlessness was reported in all three studies; adjusted OR 3.5 (95% CI: 3.0–4.1) [28^▪▪^], adjusted OR 2.7 (95% CI: 1.8–4.2) [27^▪^] and RR 2.0 (95% CI: 1.7–2.2) [26^▪^]. Being overweight was also associated with breathlessness but not as strongly as obesity (adjusted OR 1.5; 95% CI: 1.4–1.7) [28^▪▪^].

Adjustment for level of physical fitness, measured using a submaximal cycle test showed an independent association between obesity and breathlessness [27^▪^]. This suggests that the obesity itself has an impact on breathlessness regardless of physical fitness [27^▪^]. Obesity was also strongly associated with breathlessness after adjusting for FVC, adjusted OR 3.1 (95% CI: 2.6–3.6) [27^▪^]. An interaction was found between higher BMI and lower lung volumes (FVC) – the increase in breathlessness with increasing BMI was steeper in people with smaller lung volumes (compared with people with larger lungs and airways) [28^▪▪^]. The study found that the higher breathlessness prevalence among obese women than in obese men was related to the smaller lung volumes in women [28^▪▪^].

### Overlap and interplay of conditions

Only two studies specifically evaluated the co-existence and level of overlap of underlying contributing conditions in people with breathlessness [25,27^▪^], and a third study had data on overlap of COPD and heart failure only [25]. Almost two-thirds (67–69%) of individuals with breathlessness reported two or more concurrent underlying conditions that were likely to contribute to breathlessness [26^▪^,27^▪^], and approximately 14% had both a respiratory condition and a heart condition [26^▪^]. COPD and heart failure were concurrently present in 14% of breathless individuals [25].

The association between a condition and breathlessness were mostly evaluated with logistic regression models [21,22^▪^,23^▪^,27^▪^,^28^^▪▪^,^29^] or chi-squared test [24,26^▪^]. Two studies also evaluated the interaction of conditions on breathlessness when two (or more) conditions overlapped, by adding a second condition to the logistic regression models: between FVC and BMI [28^▪▪^] and between anxiety and depression [23^▪^]. There was a lack of studies using more complex analyses to evaluate the interplay between overlapping conditions and breathlessness. Even though the use of machine learning as a method in health research has seen a rapid increase lately in other fields [19], we could not identify any studies using machine learning to analyse underlying conditions contributing to the presence and severity of breathlessness.

## RESEARH GAPS AND IMPLICATIONS FOR FURTHER RESEARCH

The task of assessing underlying conditions among breathless individuals in the general population is challenging. There is a delicate interplay of many different factors such as multiple different diseases and conditions simultaneously present, different mental states, hereditary factors and social and environmental factors that could be related to breathlessness. To assess all of these in single studies is a daunting, but very important task.

Although this review focused on the general population, in the authors’ opinion, data on comorbid conditions contributing to breathlessness in disease-specific populations (such as patients with asthma, COPD, or heart failure) is likely even more scarce. There is a need for studies across different populations, including people with severe illness(es) and high levels of breathlessness, the elderly, those receiving palliative care and in people at the end of life.

Only a few studies examining underlying conditions among breathless individuals in a general population were found in this review. Most studies evaluated the presence of underlying conditions using self-report of physician diagnosed disease by the participants, which ultimately affects the certainty of the results. Studies using objective measures such as spirometry, heart ultrasound, computed tomography, blood samples and other are needed to get the full and more valid picture of underlying disease that may be undiagnosed. Anxiety, depression, and angina pectoris has, in this review, been assessed using different short questionnaires or simple questions which might give rise to inaccurate results and possible over diagnosis. Studies using validated and more in-depth measures for the diagnosis of these conditions are needed. As anxiety and depression often coexist it is not possible, based on available studies, to establish which of these conditions has the most influence on breathlessness. Methods to study the separate association from each condition are needed.

Multiple conditions are plausible contributors to breathlessness and are often overlapping with complex interplays. Level of overlap and comorbidity is associated with increased breathlessness as discussed in this review but more data is needed on the subject [1]. Machine learning could be an alternative to the statistical regression models used in many of the studies in this article. The use of machine learning could aid the analysis of associations and interactions between multiple factors [20], such as of interactions between different heart and lung diseases, BMI, anxiety and depression in regard to breathlessness. Machine learning could also be used to identify and rank the importance of the multiple underlying conditions among breathless individuals in the general population.

There is scarce information available assessing the impact of different underlying conditions among breathless individuals on other outcomes of interest. One study stated that underlying heart conditions had more negative impact on quality of life than underlying respiratory conditions [22^▪^]. This data is interesting, but more research is needed on this topic to put the importance of underlying conditions into perspective. More studies are also needed examining the impact of the presence of different underlying conditions and multimorbidity on relevant outcomes such as healthcare utilization, quality of life and survival.

There is only low-quality data on the association between heart failure and breathlessness according to results from studies included in this review. Larger and more adequate studies are needed to establish the role of heart failure on breathlessness in the general population.

Only unidimensional tools have been used to assess breathlessness in the studies included in this review. In future studies, there is a need to assess presence of breathlessness using multidimensional methods.

Recall of breathlessness is a different entity than experienced breathlessness and the two entities might be related to different underlying conditions [30]. This issue has not been addressed previously and studies examining the actual breathlessness using exercise testing such as bicycle test, 6-min walking test or others are needed. This type of information would give interesting new perspectives that could be used clinically.

## CONCLUSION

Breathlessness is common in the general population and is associated with several underlying conditions. Respiratory conditions are the most common underlying condition but more studies with refined methods such as machine learning are needed to examine the complex interplay between breathlessness and conditions. The impact from different underlying conditions on other relevant outcome measures such as quality of life and survival are needed.

## Acknowledgements

*None.*

### Financial support and sponsorship

*J.S. was supported by an unrestricted grant from the Scientific Committee of Blekinge County Council. M.E. and M.O. was funded by an unrestricted grant from the Swedish board of science (ref. 2019-02081).*

### Conflicts of interest

*There are no conflicts of interest.*

## REFERENCES AND RECOMMENDED READING

Papers of particular interest, published within the annual period of review, have been highlighted as:

▪ of special interest
▪▪ of outstanding interest
